# Co-crystallization of atomically precise metal nanoparticles driven by magic atomic and electronic shells

**DOI:** 10.1038/s41467-018-05584-9

**Published:** 2018-08-22

**Authors:** Juanzhu Yan, Sami Malola, Chengyi Hu, Jian Peng, Birger Dittrich, Boon K. Teo, Hannu Häkkinen, Lansun Zheng, Nanfeng Zheng

**Affiliations:** 1 State Key Laboratory for Physical Chemistry of Solid Surfaces, Collaborative Innovation Center of Chemistry for Energy Materials, and National & Local Joint Engineering Research Center for Preparation Technology of Nanomaterials, College of Chemistry and Chemical Engineering, Xiamen University, 361005 Xiamen, China; 2 Departments of Physics and Chemistry, Nanoscience Center, University of Jyväskylä, FI-40014 Jyväskylä, Finland; 3 Heinrich-Heine Universität Düsseldorf, Anorganische Chemie und Strukturchemie, Universitätsstrasse 1, Gebäude 26.42.01.21, 40225 Düsseldorf, Germany

## Abstract

This paper reports co-crystallization of two atomically precise, different-size ligand-stabilized nanoclusters, a spherical (AuAg)_267_(SR)_80_ and a smaller trigonal-prismatic (AuAg)_45_(SR)_27_(PPh_3_)_6_ in 1:1 ratio, characterized fully by X-ray crystallographic analysis (SR = 2,4-SPhMe_2_). The larger cluster has a four concentric-shell icosahedral structure of Ag@M_12_@M_42_@M_92_@Ag_120_(SR)_80_ (M = Au or Ag) with the inner-core M_147_ icosahedron observed here for metal nanoparticles. The cluster has an open electron shell of 187 delocalized electrons, fully metallic, plasmonic behavior, and a zero HOMO-LUMO energy gap. The smaller cluster has an 18-electron shell closing, a notable HOMO-LUMO energy gap and a molecule-like optical spectrum. This is the first direct demonstration of the simultaneous presence of competing effects (closing of atom vs. electron shells) in nanocluster synthesis and growth, working together to form a co-crystal of different-sized clusters. This observation suggests a strategy that may be helpful in the design of other nanocluster systems via co-crystallization.

## Abstract (web summary)

Atomic shell closure and electronic shell closure are generally considered to be competing effects in stabilizing magic-sized metal nanoclusters. Here, the authors show, by co-crystallizing two differently-sized clusters, that both mechanisms can work concurrently during magic cluster synthesis.

## Introduction

Atomically precise metal nanoparticles (also called metal nanoclusters) stabilized by organic ligands are of interest due to their intermediate size that bridges atoms and bulk solids, inducing distinct physico-chemical properties in the quantum-size regime^1–4^. In recent years, there has been explosive advances in building various nanoarchitectures via a wide range of synthetic strategies^5^. However, factors driving the thermodynamic stability of discrete nanometer sizes are largely unknown^6,7^.

Ligand-stabilized metal nanoclusters are typically produced in solution by reducing metal salts in the presence of protecting ligands. Achieving a high degree of control over their size and atomic composition is generally a complex process that is affected by several factors during synthesis such as the metal:ligand ratio, reaction temperature, rate of reduction, post-processing methods such as size focusing, and purification. A general view is that the formation of the protected clusters can be stopped in the process of either nucleation or etching once a magic size and composition is reached^3^. These magic sizes and compositions are then entities trapped in local free-energy minima surrounded by kinetic barriers in the multi-dimensional phase space. Three factors are commonly considered to play important roles in the formation of magic cluster sizes and compositions: (i) Favorable surface chemical structure (the ability to protect the metal core from the environment); (ii) Favorable atomic packing, often seen as spherical structures of concentric polyhedral shells; (iii) Closed-shell electronic structure stabilizing smaller, molecule-like nanoclusters through opening of a large energy gap between the highest occupied molecular orbital (HOMO) and the lowest unoccupied molecular orbital (LUMO), the so-called HOMO-LUMO gap. The process has thus certain analogies to the formation of magic metal nanoclusters in gas-phase experiments, where the crucial roles of electronic shells and atomic packing (atomic shells) have been discussed extensively since the 1980s^8–12^.

In this work, we present and analyze direct evidence that the formation of both atomically closed-shell and electronically closed-shell nanoclusters is possible concurrently in a synthesis that produces atomically closed-shell intermetallic (AuAg)_267_(2,4-SPhMe_2_)_80_ nanoparticles (AuAg)_267_ and electronically closed-shell (AuAg)_45_(2,4-SPhMe_2_)_27_(PPh_3_)_6_ nanoclusters (AuAg)_45_. These species were identified from a co-crystal having an unusual packing motif for metal nanoparticles. The co-crystallization of two such different-sized nanoparticles, stabilized by competing mechanisms (atomic vs. electronic shell effects) to achieve magic sizes and compositions suggests a valid strategy that might be helpful in the synthesis and co-crystallization of other bimodal nanocluster systems.

## Results and Discussion

### Synthesis and characterization

**Fig. 1:**
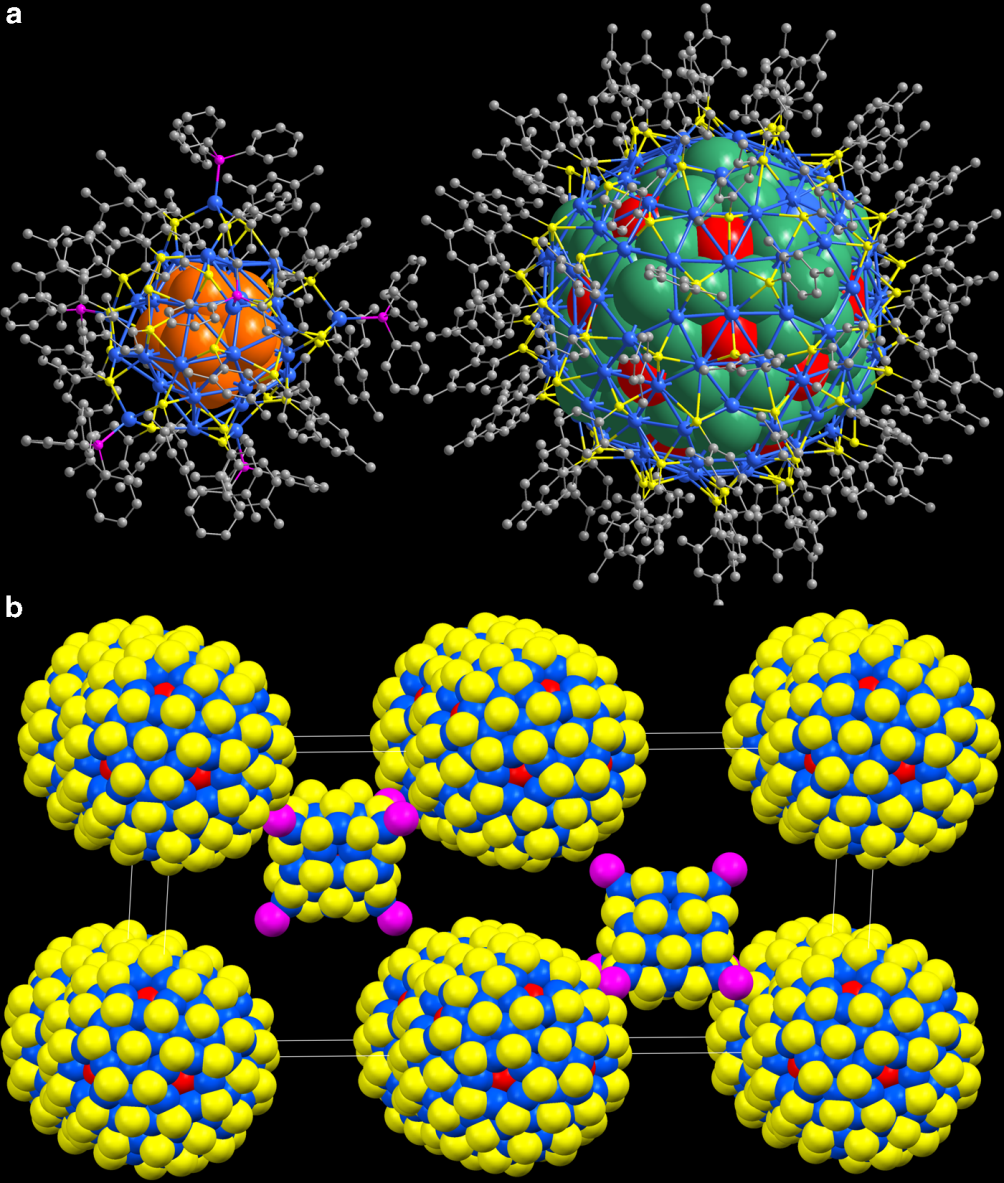
Cocrystal structure of (AuAg)_267_∙(AuAg)_45_. **a** The total structure of the plasmonic (AuAg)_267_ nanoparticle co-crystallized with the molecule-like (AuAg)_45_ cluster. All hydrogen atoms are omitted for clarity. **b** Unit cell of the three-dimensional structure of spherical (AuAg)_267_ nanoparticles and trigonal prismatic (AuAg)_45_ nanoclusters in space-filling model. All hydrogen and carbon atoms are omitted for clarity. Color code: orange Au, sea green and red AuAg, blue Ag, yellow S, magenta P, gray C

The mixture of (AuAg)_267_ and (AuAg)_45_ nanoclusters was prepared by reducing the metal precursors AuPPh_3_Cl and AgNO_3_ (1:1 ratio) with NaBH_4_ in the presence of 2,4-dimethylbenzenethiol (HSR), triphenyphosphine (PPh_3_), tetraphenylphosphonium bromide (PPh_4_Br), and triethylamine in methanol/dichloromethane at 0 °C (see SI for more details). Transmission electron microscopic (TEM) analysis revealed that the as-prepared Au–Ag compounds were dominantly in two sizes, nearly 50/50, for ~2.48 nm and ~1.10 nm (Supplementary Fig. 1). To determine their detailed molecular structure, much effort was devoted to crystallize the as-prepared products into dark single crystals (Supplementary Fig. 2) suitable for X-ray diffraction by slowly diffusing hexane into the dichloromethane solution at 0 °C. The X-ray single-crystal structure analysis revealed the co-crystallization of both (AuAg)_267_ and (AuAg)_45_ nanoclusters (Fig. 1a). The (AuAg)_267_ nanoparticle is spherically shaped and the (AuAg)_45_ nanocluster is a trigonal prism. These two differently shaped nanoclusters are hierarchically assembled in a hexagonal *P*6_3_/*m* space group in a 1:1 ratio (Fig. 1b, Supplementary Figs. 3 and 4).

### Atomic structure

**Fig. 2:**
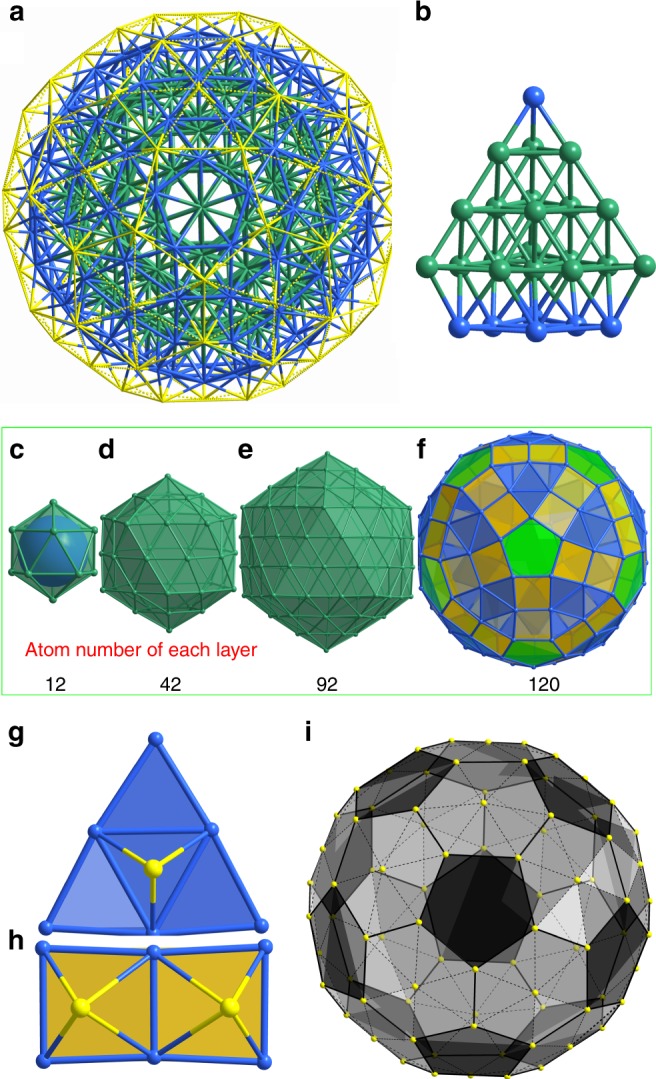
Representative molecular structure of the plasmonic (AuAg)_267_ nanoparticle. **a** Buckyball-shape (AuAg)_267_ nanoparticle. **b** Layered ABCAC packing (or ABCB if the central Ag atom is ignored) of metal atoms. **c**–**f** Anatomy of metal framework of (AuAg)_267_ nanoparticle: the first Mackay icosahedral shell (*ʋ*_1_) with 12 M atoms (**c**); the second Mackay icosahedral shell (*ʋ*_2_) with 42 M atoms (**d**); the third Mackay icosahedral shell (*ʋ*_3_) with 92 M atoms (**e**); and the fourth anti-Mackay shell (*ɷ*_4_) with 120 Ag atoms (**f**). **g** Three-fold coordinated thiolate centered on six-Ag-atom triangle (highlighted in blue in panels (**f**) and (**g**). **h** Two tetradentate thiolates capped on two edge-shared squares (highlighted in yellow in panels (**f**) and (**h**). **i** Sulfur atoms (yellow) arranged in a slightly distorted truncated icosahedron (buckyball). All hydrogen and carbon atoms are omitted for clarity. Color code: sea green and red AuAg, blue Ag, yellow S

A detailed analysis revealed that (AuAg)_267_ nanoparticle exhibits a remarkable high-symmetry three-layer Mackay icosahedron (MIC) molecular structure with efficient *fcc* atom packing (Fig. 2a, b), with the fourth layer in anti-Mackay configuration. The geometrical anatomy of the (AuAg)_267_ nanoparticle can be represented by a four-shell metal structure *υ*_0_(1)@*υ*_1_(12)@*υ*_2_(42)@*υ*_3_(92)@*ω*_4_(120), which is protected by a buckyball-like S_80_ thiolate-ligand shell. This “shell-by-shell” or “Matryoshka doll” representation is depicted in Fig. 2c–f. The 120 atoms in the fourth shell are mutually connected to form a semiregular polyhedron containing 20 *υ*_2_ triangles, 60 squares, and 12 pentagons. The center of each *υ*_2_ triangular and square face in the fourth shell is capped by a thiolated ligand (Fig. 2g, h), such that the 80 sulfur atoms from the thiolated ligands define a slightly distorted buckyball (Fig. 2i). A more detailed description of the (AuAg)_267_ structure is found in the SI text (Supplementary Fig. 5 and Supplementary Table 3). The (AuAg)_45_ cluster has a trigonal prismatic shape, containing a two-shell tri-capped-prism metal framework and protected by 27 thiolates and 6 triphenylphosphines (see Supplementary Discussion, Supplementary Figs. 6 and 7). The molecular structure is almost identical to Au_9_Ag_36_(SPhCl_2_)_27_(PPh_3_)_6_ reported by us previously^13^.

Mackay introduced the concept of the hierarchical icosahedral structure 50 years ago^14^. The cluster of 55 atoms within the second icosahedral shell occurs frequently, as determined by mass spectroscopy or theoretically forecasted, and thus was often referred to as Mackay icosahedron. In the past decades, the two-layer M_55_ Mackay icosahedra^14^ have also been disclosed in a diversity of nanosized noble metal clusters via single-crystal x-ray diffraction, namely, a three-shell 145-metal-atom Pd_145_(CO)_*x*_(PEt_3_)_30 _(*x* ≈ 60) cluster^15^, a Pd-Pt four-shell 165-metal-atom (*μ*_12_-Pt)Pd_164-*x*_Pt_*x*_(CO)_72_(PPh_3_)_20_ (*x* ≈ 7) cluster^16^ and 133-metal-atom Au_133_(SR)_52_ one^17,18^, as well as a two-shell 55-metal-atom Pd_55_(^*Pi*^Pr_3_)_12_(μ_3_-CO)_20_ cluster^19^, which were of particular significance as revelatory for predicting the structures of these air-sensitive 55-metal-atom M_55_L_12_Cl_x_ (M = Au, Pd, Rh, or Ru; *x* = 6 or 20) clusters reported by Schmid et al.^20,21^. In the present work, the next Mackay overlayer based on icosahedral M_55_ is crystallographically observed in (AuAg)_267_, giving rise to well-known three complete Mackay metal shells of 147 atoms^14^, *υ*_0_(1)@*υ*_1_(12)@*υ*_2_(42)@*υ*_3_(92), at variance to embryonic growth of decahedral (Au_102_^22^, Au_130_^23^, Au_246_^24^, Ag_136_, and Ag_374_^25^) and *fcc* cuboctahedral Au_279_^26^ clusters.

### Distribution of Au and Ag

Considering the positional disorder of metal atoms might occur in the kernels of (AuAg)_267_ nanoparticle and (AuAg)_45_ nanoclusters, we have used different methods to get insight into their metallic distributions. The outermost metallic shells of (AuAg)_267_∙(AuAg)_45_ co-crystal consist of Ag atoms which are in sync with the coordination modes of Ag-SR and Ph_3_P-Ag-SR in ways analogous to previous works^27–30^, but at odds with the typical Au_*n*_(SR)_*n*+1_ (*n* = 1 or 2) staple motifs commonly observed in many thiolated gold clusters^22,31,32^. As shown in Table 1, the approximate composition of the molecular formula in term of central Au (Ag) and Ag (Au) atoms in the inner shells (Au atoms for the (AuAg)_45_ cluster and Ag for the (AuAg)_267_ nanoparticle) was established from Au_*x*_Ag_1-*x*_ occupancies based upon least-squares refinement of the X-ray data. The total crystallographically estimated contribution of *x* = 93.8 for Au_*x*_Ag_312-*x*_ (2.33:1 molar ratio of Ag/Au for total 312 metallic atoms in (AuAg)_267_∙(AuAg)_45_) tallied with that found from an inductively coupled plasma mass spectrometric (ICP-MS) Ag/Au determination (2.36:1 molar ratio of Ag/Au for a total of 312 metallic atoms in (AuAg)_267_∙(AuAg)_45_), as well as the outcome of an energy dispersive X-ray spectroscopic (EDS) analysis (2.36:1 molar ratio of Ag/Au for total 312 metallic atoms in (AuAg)_267_∙(AuAg)_45_). Interestingly, there are some regularities in the Au–Ag distribution in (AuAg)_267_∙and (AuAg)_45_ as described below.

**Table 1:** Au/Ag ratio in (AuAg)_267_·(AuAg)_45_ characterized by various methods

Method	Ag/107	Au/197	Molar(Ag)	Molar(Au)	Ag:Au molar ratio
ICP-MS	139.22	108.53	1.30	0.55	2.36:1
EDS	—	—	0.702	0.298	2.36: 1
X-ray	—	—	218.2	93.8	2.33: 1

For (AuAg)_267_, the central site has 100% Ag occupancy, and all sites on *C*_3_ axes of the 147-atom icosahedral kernel are Ag-rich (Ag occupancies >85%), and the remaining sites of the first (υ_1_) and second (υ_2_) shells are disordered with roughly 50-50 occupancies of Au and Ag. The same segregation phenomenon occurs in the third (υ_3_) shell wherein the Ag-rich (90–100%) atoms on 3-fold axes are surrounded by Au-rich (70–85%) atoms on the vertices and the edges. Despite these seemingly random disorder (note that Au and Ag form completely disordered solid solutions), a careful examination of the relative Au:Ag occupancies revealed some regularities, as follows. The first three shells, along with the central atom (υ_0_) and the anti-Mackay-like surface shell (ω_4_), exhibit alternating Ag- and Au-rich shells (average occupancies in parentheses): *υ*_0_(100% Ag)@*υ*_1_(ca. 60% Au)@*υ*_2_(ca. 60% Ag)@*υ*_3_(ca. 80% Au; ca. 90% Ag on 3-fold axes)@*ω*_4_(100%Ag) (see Supplementary Fig. 8 and CCDC-1839942). This alternating shell behavior maximizes the Au–Ag heteronuclear bonds at the expenses of homonuclear Au–Au and Ag–Ag bonds due to the disparity in the electronegativity of Au and Ag, which increases the bond energy due to the ionic character of the heteronuclear bonds^33^. DFT calculated results also suggested that Ag is systematically positively charged and Au negatively charged in (AuAg)_267_ (Supplementary Table 4), so there is always a slight electron transfer from Ag to Au. Unlike (AuAg)_267_, the 9 atoms in M_9_ tricapped trigonal prism core of the (AuAg)_45_ cluster, are simply composed Au with occupancies nearly 100% (see CCDC-1839941).

### Interparticle assembly

**Fig. 3:**
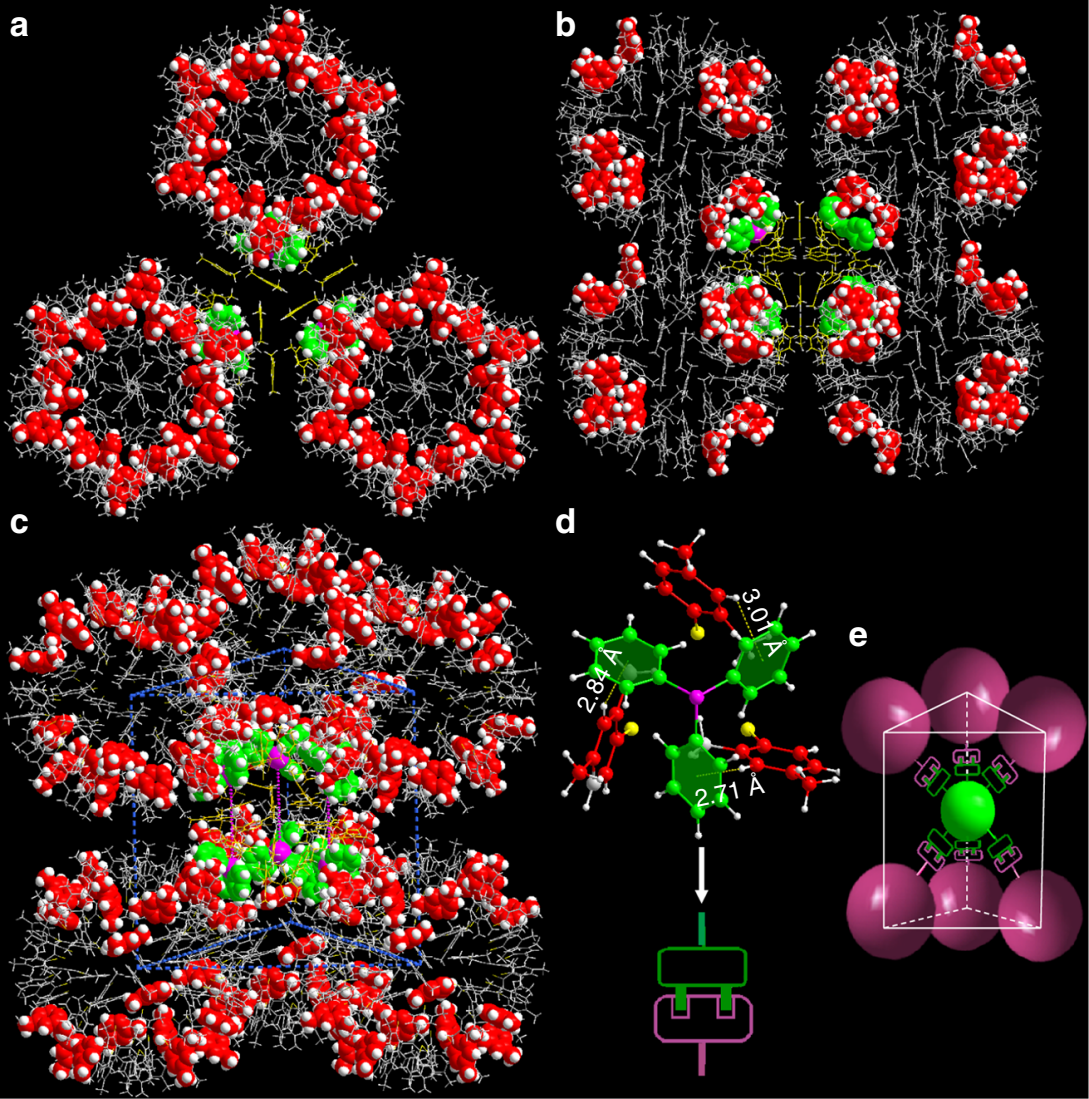
Interparticle self-assembly directed by the ligand anisotropy of surface patterns. **a**–**c** Arrangement of surface ligands of both (AuAg)_267_ and (AuAg)_45_ in the crystal lattice: top view (**a**) and side view (**b**) and (**c**). **d** The C-H⋅⋅⋅π interactions among the interparticle ligands, similar to tightly jointed plugs and sockets. **e** Scheme showing directional packing of binary composites via anisotropic ligand surface. Color code: magenta P, gray, red, gold, and green C, white H

Apart from the self-organized process for the formation of individual nanoparticles at the atomic level, (AuAg)_267_ and (AuAg)_45_ components serve as building blocks, hierarchically assembled into a three-dimensional structure with a relative arrangement similar to PtB or anti-NiAs structures (Fig. 1b and Supplementary Figs. 9–11). In the crystal lattice, each (AuAg)_267_ is surrounded by six octahedral-arranged (AuAg)_45_ nanoclusters while each (AuAg)_45_ is neighbored by six (AuAg)_267_ nanoparticles arranged in a trigonal prismatic fashion. The particle-to-particle distance is 2.78 nm. The effective size ratio *γ* = *R*_small_/*R*_large_ of nanoparticles that are arranged in the PtB-type superlattice is usually in the range of ~0.4. However, structural analysis indicate that the sizes of (AuAg)_267_ and (AuAg)_45_ are 1.65 nm (Supplementary Fig. 3) and 1.05–1.25 nm (Supplementary Fig. 4), respectively (0.636 ≤ *γ* ≤ 0.757)^34^. The values 0.636–0.757 significantly exceed the size ratio predicted for a stable PtB-type structure. To show why such interactions do not lead to more closely packed CsCl or AlB_2_-type structures that entropy alone would favor, we focused on the rigid conformation and specific molecular shape of the particles’ surface layer. As mentioned above, the (AuAg)_267_ nanoparticle has a geometrically-isotropic spherical structure whose thiolated ligands are nearly homogeneously arranged on its metal core. (AuAg)_45_ particle is coordinated by two different ligands which are anisotropically distributed. As shown in Fig. 3a–c, each (AuAg)_45_ nanoparticle has six nearest (AuAg)_267_ neighbors coincide well with the directions of the six phosphine ligands of (AuAg)_45_ arranged in a trigonal prismatic fashion. The parallel thiolated ligands of (AuAg)_45_ are situated on the crack between two neighboring (AuAg)_267_ particles. In this way, the surface symmetry of two different nanoparticles is perfectly matched. The interacting ligands (phosphines of (AuAg)_45_ and thiolates of (AuAg)_267_) between diblocks are akin to “socket-plug” combination via C-H⋅⋅⋅π interactions (Fig. 3d, e). The clustering of different ligands on the periphery of diblocks leads to packing constraints and further generates the intriguing and unconventional PtB-type structure. The cocrystalline superlattice of (AuAg)_267_ and (AuAg)_45_ provides a system suitable for engineering hierarchical structures and also offers insights into the assembly formation mechanism at the atomic level. Both the incommensurate interactions and shape disparities are untangled during their self-organization.

### Optical properties

**Fig. 4:**
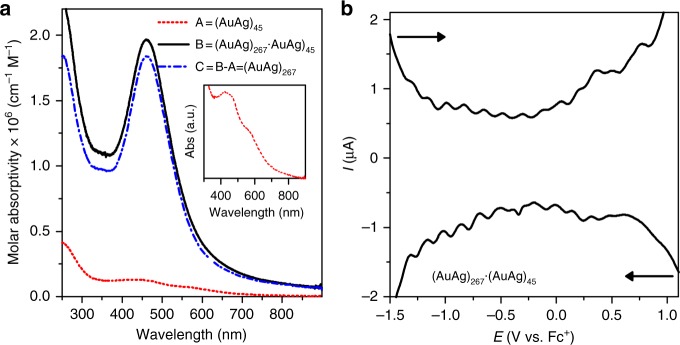
Optical and electrochemical properties of (AuAg)_267_∙(AuAg)_45_. **a** Molar absorptivity spectra of (AuAg)_267_∙(AuAg)_45_ cocrystal, plasmonic (AuAg)_267_, and molecule-like (AuAg)_45_ clusters dissolved in CH_2_Cl_2_. Inset plot is the enlarged view of UV-Vis spectra of (AuAg)_45_. **b** DPV curves of (AuAg)_267_∙(AuAg)_45_ cocrystal dissolved in CH_2_Cl_2_

Figure 4a shows the UV-vis absorption spectra for (AuAg)_267_ alloy nanoparticles with (AuAg)_45_ content, as well as the two corresponding independent species. Only one plasmon band at 460 nm is observed in (AuAg)_267_∙(AuAg)_45_ CH_2_Cl_2_ solution. The distinct difference between the (AuAg)_267_ nanoparticle and the (AuAg)_45_ nanocluster was demonstrated by comparing their absorption spectra. The (AuAg)_45_ nanocluster has a structured absorption spectrum with absorption bands at 434 and 570 (shoulder) nm, behaving as small molecules rather than as typical metallic nanoparticles. The visible absorption of the co-crystallized compound in CH_2_Cl_2_ mainly arises from the surface plasmon band of 267 Au–Ag alloyed composite. The dominance of the plasmon absorption band of (AuAg)_267_ nanoparticles in the dissolved 1:1 co-crystal solution is due to the large discrepancy in extinction coefficients between the large and small clusters. Their molar absorptivity difference at 532 nm was almost a multiple of ten in CH_2_Cl_2_ (Supplementary Fig. 12).

### Electronic structure

**Fig. 5:**
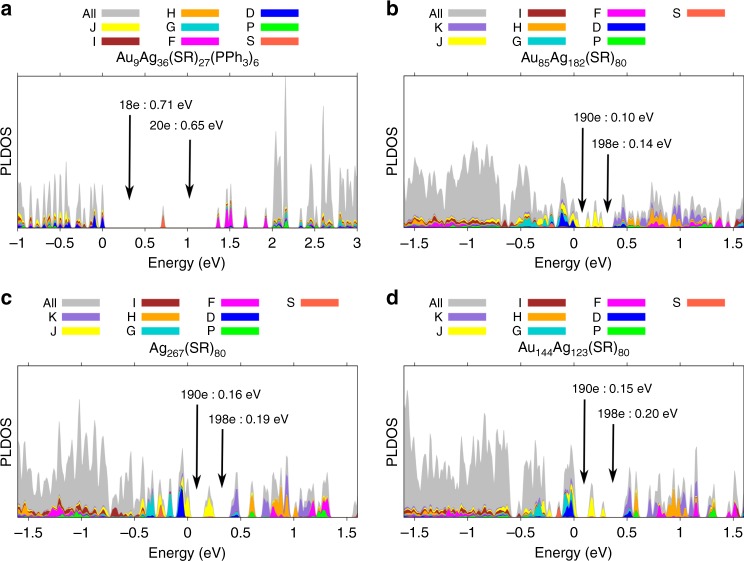
Calculated electronic structures of (AuAg)_267_ and (AuAg)_45_. **a** Projected Local Density of States (PLDOS) of the (AuAg)_45_ cluster. **b**–**d** The same analysis for a hypothetical all-silver Ag_267_ and for two intermetallic clusters with compositions of Au_85_Ag_182_ and Au_144_Ag_123_, all computed in the nanoparticle observed crystal structure of (AuAg)_267_. The various colors denote the weights of orbitals projected onto spherical harmonics, centered at the cluster center-of-mass. The energy of the highest occupied molecular orbital (HOMO) is at zero. The major energy gaps around or above the HOMO energy are indicated by arrows

We analysed the electronic structure of both nanoparticles using the density functional theory (DFT, for details see Methods). The count of free metallic electrons in the neutral (AuAg)_267_ cluster is 267–80 = 187^35^. Due to its near-spherical core shape, it is reasonable to compare the electronic structure of the metal core to the simplified spherical jellium model that describes quantization of electrons in a uniform electron gas trapped in an infinitely deep, spherical potential well^36^. This model predicts a major energy gap at 186 electrons, in a shell configuration of 1S^2^ 1P^6^ 1D^10^ 2S^2^ 1F^14^ 2P^6^ 1G^18^ 2D^10^ 1H^22^ 3S^2^ 2F^14^ 1I^26^ 3P^6^ 1J^30^ 2G^18^, and weaker energy gaps at 196 and 198 electrons, after completion of 3D^10^ and 4S^2^ shells, respectively. However, the calculated projected local density of electron states (PLDOS) of the three considered models for the (AuAg)_267_ cluster do not show an energy gap at 186 electrons, rather there are two well-defined gaps at 190 electrons (0.10–0.16 eV) and 196 electrons (0.14–0.20 eV) (Fig. 5). The analysis of the PLDOS data of (AuAg)_267_ clusters shows two deviations from the ideal shell order from the spherical jellium model: (i) both 3D^10^ and 4S^2^ shells are lowered in energy below HOMO, i.e., have become fully occupied, and at the same time (ii) the 1 J shell is widely broadened and fragmented on both sides of the HOMO energy, i.e., several 1J-symmetric states that are fully occupied in the spherical jellium model are now shifted upwards above the HOMO and are unoccupied. This re-organization of the shell structure is likely due to the lowering of symmetry from the perfect sphere by the discrete point-group symmetry of the core, as well as of the ligand layer. The lack of a detectable HOMO-LUMO energy gap demonstrates that the electronic structure does not provide a leading mechanism to stabilize the observed atomic structure of (AuAg)_267_, hence, the stability must arise from the geometrical concentric packing of metal atoms to magic Mackay/anti-Mackay icosahedral shells and the structure of the protecting ligand shell, as discussed in detail above.

In contrast, the analysis of the electronic structure of the smaller (AuAg)_45_ cluster (Fig. 5) shows a clear HOMO-LUMO energy gap (0.71 eV) as expected for 18 jelliumatic electrons (45–27 = 18). The projection to angular momentum components indicates the closing of the 1D^10^ shell at 18 electrons, as expected, and another major energy gap (0.65 eV) at 20 electrons, i.e., after closing the 2S^2^ shell. This cluster is thus clearly energetically stabilized by the electron shell closure at 18 electrons, i.e., at a magic electron number. The calculated UV-vis absorption spectrum (Supplementary Fig. 13) agrees qualitatively with the measured one, showing a maximum at around 475 nm and a shoulder around 580 nm, slightly red-shifted from the experimental values of 434 nm and 570 nm (Fig. 4a, inset), respectively.

### Electrochemical properties

A typical differential pulse voltammetric (DPV) response for the as-prepared (AuAg)_267_∙(AuAg)_45_ co-crystal dissolved in 0.1 M Bu_4_NPF_6_/CH_2_Cl_2_ at room temperature, exhibiting 14 evenly spaced peaks (Δ*V* is 0.16 eV on average) characteristic of charge injection to the metal cores is given in Fig. 4b, which is a clear confirmation that (AuAg)_267_∙(AuAg)_45_ are indeed multivalent redox species. We did not observe a clear HOMO-LUMO gap among quantized double layer charging (QDL) reduction and oxidation peaks. Our DFT calculations support this observation, given that it is likely that the larger nanoparticle dominates the redox behavior. As shown in Supplementary Fig. 14, the dense spacing of electronic states of (AuAg)_267_ around the HOMO yields a quadratic behavior of the total energy of the cluster *E* as a function of charge *Q*, *E* = *aQ*^2^ + *bQ* + *c*, akin to the charging of a classic metallic sphere. From the parabolic fits (Supplementary Fig. 14), a constant charging energy (*d*^2^*E*/*dQ*^2^ = 2*a*) can be evaluated according to the standard theory of metallic quantum dots^37^. The calculated constant charging energy (in vacuum) for the three considered models of (AuAg)_267_ is about 1.02 eV per reduction/oxidation step, which indicates that the effective dielectric constant of the electrolyte is about 6.4, estimated as the ratio between the calculated (in vacuum) and measured (in solution) charging energy. The case of (AuAg)_45_ is interesting, as the HOMO-LUMO energy gap *E*_HL_ ≅ 0.7 eV and the cumulative gap at 20 shell electrons are both visible in the calculation of *E(Q)*, see Supplementary Figs. 14 and 15. Hence, the calculations predict that (AuAg)_45_ should contribute just a few discrete charging peaks in the energy (voltage) region probed by the DPV experiment. The exact locations of those charging peaks are undetermined since the relative redox potentials of the larger and smaller particles are not known. However, we note that the experimental DPV data of (AuAg)_267_∙(AuAg)_45_ indeed is more complex than the data from Ag_206_ that we reported recently^38^, which may result from the presence of two particles having distinctly different redox behaviors.

In conclusion, the co-crystallization of a giant (AuAg)_267_ nanoparticle and a much smaller (AuAg)_45_(2,4-SPhMe_2_)_27_ cluster into multicomponent PtB-type hierarchical structures has been discovered. The present report exemplifies a bimodal particle nucleation and growth from solution driven by geometrically closed-shell and electronically closed-shell effects. Specifically, the plasmonic (AuAg)_267_ nanoparticle adopts a magic shell packing, that is, a four concentric core-shell structure of the Ag@M_12_@M_42_@M_92_@Ag_120_(SR)_80_ (M = Au or Ag) with an idealized *I*_*h*_ symmetry, while molecular (AuAg)_45_ cluster satisfies an 18-electron superatom shell closure. The 147-metal-atom solid core in (AuA)_267_ possesses a magic number of atoms, which was unprecedented in metal nanocluster family. Meanwhile, a well-organized metal segregation phenomenon, alternating Ag- and Au-rich shells behavior, was observed in the (AuAg)_267_ metal framework. Besides, we analyzed the packing constraint of the (AuAg)_267_∙(AuAg)_45_ co-crystal and its relation to surface ligand structures, which was helpful for the understanding of clustering process in unconventional PtB-structure superlattices.

## Methods

### Synthesis of the mixture of (AuAg)_267_ and (AuAg)_45_ (**1**)

In a typical preparation, 10 mg of AgNO_3_ or 20 mg of AgSbF_6_ was dissolved in 1 ml of methanol, followed by the addition of 12 mg AuPPh_3_Cl in 4 ml of dichloromethane. The mixture was cooled to 0 °C in an ice bath, 5 μL of 2,4-dimethylbenzenethiol, 4 mg PPh_3_, and 10 mg of tetraphenylphosphonium bromide were then added. After 20 min of stirring, 1 ml of an aqueous solution of NaBH_4_ (40 mg/mL) and 50 μl of triethylamine were added quickly to the reaction mixture under vigorous stirring. The reaction mixture was aged for 12 h at 0 °C. The aqueous phase was then removed. The organic phase was washed several times with water and evaporated for further analysis. Dark single crystals suitable for X-ray diffraction study were grown by a double-layer of hexane/CH_2_Cl_2_ solution of crude product at 4 °C for two weeks (Supplementary Table 1). The yield of (AuAg)_267_∙(AuAg)_45_ was ~15% (based on Au).

### Synthesis of (AuAg)_45_ nanocluster

Same as (**1**) with the exception of increasing the usage of PPh_3_ to 8 mg. Both (AuAg)_267_∙(AuAg)_45_ (hexagonal-prismatic shape) and (AuAg)_45_ (semi-thick hexagonal plate, main product and Supplementary Table 2) crystals are obtained (Supplementary Fig. 2). The yield of (AuAg)_45_ was ~10% (based on Au).

### DFT calculations

All the atomistic DFT computations were performed using the real-space code package GPAW^39^. The experimental crystal structures of (AuAg)_45_ and (AuAg)_267_ were used as such for further analysis of the electronic density of states and their projections to spherical harmonics. Three compositional models were built for the larger particle, one having a hypothetical all-silver Ag_267_ core and two intermetallic cores Au_85_Ag_182_ and Au_144_Ag_123_. The compositions for the intermetallic clusters were selected to represent the absolute maxima and minima in integrated Au occupation, based on the probabilities (partial occupation numbers) of these metals in the refined crystal structure data. To study the charging behavior, the total energy of the clusters was evaluated as a function of charge via single-point calculations, i.e., ignoring the structural relaxation. The electron–electron interactions were described by the PBE-functional and the PAW setups for silver and gold included relativistic effects. The real-space grid spacing was 0.25 Å. Optical spectra were calculated by using the linear responsetime-dependent DFT module implementedin the GPAW software.

### Data availability

The X-ray crystallographic coordinates for structures reported in this work (see Supplementary Tables 1 and 2) have been deposited at the Cambridge Crystallographic Data Centre, under deposition number CCDC-1839941 and 1839942. These data can be obtained free of charge from the Cambridge Crystallographic Data Centre via http://www.ccdc.cam.ac.uk/data_request/cif. All other relevant data are available from the corresponding authors.

## Electronic supplementary material

Supplementary Information

Peer Review File
