# Supplementary material for: Co-crystallization of atomically precise metal nanoparticles driven by magic atomic and electronic shells
Source: Nat Commun. 2018 Aug 22;9:3357. doi: 10.1038/s41467-018-05584-9 (PMC6105650; doi:10.1038/s41467-018-05584-9)
Supplement: Supplementary file 1 — Supplementary Information [file 41467_2018_5584_MOESM1_ESM.pdf]

Supplementary Methods

Raw materials. Silver nitrate ($AgNO_3$, 99%), silver hexafluoroantimonate ($AgSbF_6$, 99%), 2,4-dimethylbenzenethiol (2,4-$SPhMe_2$, 97%), tetraphenylphosphonium bromide (PPh_4Br, 99%), tetrabutylammonium hexafluorophosphate (Bu_4NPF_6, 98%) were purchased from Alfa Aesar (Tianjin, China). Sodium borohydride ($NaBH_4$, 98%), triphenylphosphine (PPh_3, 99%), triethylamine ($C_6H_{15}N$, 99.5%), dichloromethane (CH_2Cl_2, A.R.), hexane (C_6H_{14}, 99%) and methanol (CH_3OH, A.R.) were purchased from Sinopharm Chemical Reagent Co. Ltd. (Shanghai, China). The water used in all experiments was ultrapure. All reagents were used as received without further purification. (Triphenylphosphine)gold(I) chloride ($AuPPh_3Cl$) was prepared according to literature methods[1].

Synthesis of the mixture of $(AuAg)_{267}$ and $(AuAg)_{45}$ (1). In a typical preparation, 10 mg of $AgNO_3$ or 20 mg of $AgSbF_6$ was dissolved in 1 ml of methanol, followed by the addition of 12 mg $AuPPh_3Cl$ in 4 ml of dichloromethane. The mixture was cooled to 0°C in an ice bath, 5 μL of 2,4-dimethylbenzenethiol, 4 mg PPh_3 and 10 mg of tetraphenylphosphonium bromide were then added. After 20 min, of stirring, 1 ml of an aqueous solution of $NaBH_4$ (40 mg/mL) and 50 μl of triethylamine were added quickly to the reaction mixture under vigorous stirring. The reaction mixture was aged for 12 h at 0°C. The aqueous phase was then removed. The organic phase was washed several times with water and evaporated for further analysis. Dark single crystals suitable for X-ray diffraction study were grown by a double-layer of hexane/CH_2Cl_2 solution of crude product at 4°C for two weeks. The yield of $(AuAg)_{267}·(AuAg)_{45}$ was ~15%.

Synthesis of $(AuAg)_{45}$ nanocluster. Same as (**1**) with the exception of increasing the usage of PPh_3 to 8 mg. Both $(AuAg)_{267}·(AuAg)_{45}$ (hexagonal-prismatic shape) and $(AuAg)_{45}$ (semi-thick hexagonal plate) crystals are obtained (Supplementary Fig. 2). The yield of $(AuAg)_{45}$ was ~10%.

Characterizations. The UV-vis spectra were measured by Shimadzu UV-2550 Spectrophotometer with dichloromethane as solvent. High-resolution transmission electron microscopy (TEM) studies were performed on a TECNAI F30 transmission electron microscope operating at 300 kV. The samples were prepared by dropping CH_2Cl_2 solution of samples onto 300-mesh carbon-coated copper grids and immediately evaporating the solvent. Energy dispersive X-ray spectroscopy (EDX) performed with an FEI TECNAI F30 microscope operated at 300 kV. The composition of $(AuAg)_{267}$·$(AuAg)_{45}$ alloy compound was determined by inductively coupled plasma mass spectrometry (ICP-MS, Agilent 7700).

Electrochemistry. Electrochemical measurements of $(AuAg)_{267}$·$(AuAg)_{45}$ were performed with an electrochemical workstation (CHI 760e, Shanghai Chenhua Co., China) using a glass carbon working electrode (diameter 0.1 mm), a Pt counter electrode, and a SCE-reference electrode in 0.1 M Bu_4NPF_6/CH_2Cl_2. $(AuAg)_{267}$·$(AuAg)_{45}$ solutions were degassed and blanketed with a high-purity N_2 atmosphere during measurement. Ferrocene ($Fc^{0/+}$) was used as an internal reference for the SCE-reference electrode. The $Fc^{0/+}$ couple was found to be 0.554 V versus SCE in 0.1 M Bu_4NPF_6/CH_2Cl_2. All potentials in this article are reported with respect to $Fc^{0/+}$. Voltammograms of the nanoparticle solutions were acquired at 0 °C using an ice bath.

Single-Crystal X-ray Structure Analysis. $(AuAg)_{267}$·$(AuAg)_{45}$ and $Au_9Ag_{36}(SR)_{27}(PPh)_6$ (SR=2,4-dimethylbenzenethiolate) were collected on an Agilent Technologies SuperNova X-ray single-crystal diffractometer with Cu Kα radiation (λ=1.54184 Å) at 100 K. The data were processed using CrysAlisPro[2]. The structures were solved and refined using Full-matrix least-squares based on F^2 using ShelXT[3] and ShelXL[4] in Olex2[5] and Shelxle[6]. Detailed crystal data and structure refinements for both compounds are given in Supplementary Table 1 and 2.

Supplementary Discussion

crystal structure of $(AuAg)_{267}(2,4\text{-}SPhMe_2)_{80}$. The 147-atom 3-shell Mackay icosahedron in $(AuAg)_{267}$ can also be considered as 20 identical υ_3 tetrahedra that share

a common vertex (the central atom) and join together through sharing of adjacent faces (υ_3 triangles). The metal atoms in each tetrahedral motif unit are arranged in a layered ABCA manner (*fcc* stacking). In the icosahedral twinned cores of $(AuAg)_{267}$, the average metal bond length is 2.877 Å, slightly shorter than the bond distance (2.889 Å) in bulk *fcc* Ag or Au. More detailed analysis of the metal bond lengths (Supplementary Fig. 4 and Supplementary Table 3) reveals the presence of internal lattice strain distributions in the icosahedral $(AuAg)_{147}$ kernel. Namely, the interatomic spacing in the 20 equilateral outer faces is about 2~5% larger than the spacing along the radial lines connecting the 12 vertices with the former icosahedral subunit. However, a twin arrangement or anti-Mackay-like layer, denoted as $\omega_4(120)$ with 120 atoms), is adopted for fourth shell instead of a regular Mackay υ_4 icosahedral shell (with 162 atoms). The 120 atoms in 4th shell are mutually connected to form a semiregular polyhedron containing 20 υ_2 triangles, 60 squares and 12 pentagons. All thiolates are orderly distributed on the fifth shell. The center of each υ_2 triangular and square face of fourth shell is capped by a thiolated ligand, such that the 80 sulphur atoms from the thiolated ligand define a slightly distorted buckyball. Notably, 20 additional sulphur atoms occupy the 20 centers of hexagonal face of buckyball, respectively. As a result, the fifth buckyball ligand layer contains 60+20=80 sulphur atoms.

Supplementary Table 1. Crystal data and structure refinement for $(AuAg)_{267}·(AuAg)_{45}$.

Identification code	$(AuAg)_{267}·(AuAg)_{45}$
Empirical formula	$C_{964}H_{1053}Ag_{218.23}Au_{93.77}P_6S_{107}$
Formula weight	58513.27
Temperature/K	102(3)
Crystal system	hexagonal
Space group	$P6_3/m$
a/Å	36.7480(8)
b/Å	36.7480(8)
c/Å	72.1336(14)
α/°	90
β/°	90
γ/°	120
Volume/Å^3	84360(4)
Z	2.00004
ρ_{calc} g/cm^3	2.304
μ/mm^{-1}	36.164
F(000)	52853.0
Crystal size/mm^3	0.5 × 0.1 × 0.1
Radiation	Cu Kα (λ = 1.54184Å)
2θ range for data collection/°	6.662 to 122.336
Index ranges	$-41 \leq h \leq 37$, $-41 \leq k \leq 31$, $-81 \leq l \leq 30$
Reflections collected	169823
Independent reflections	43618 [R_{int} = 0.0762, R_{sigma} = 0.0709]
Data/restraints/parameters	43618/1082/1958
Goodness-of-fit on F^2	1.056
Final R indexes [$I \geqslant 2\sigma$ (I)]	R_1 = 0.0730, wR_2 = 0.2168
Final R indexes [all data]	R_1 = 0.1288, wR_2 = 0.2749
Largest diff. peak/hole / eÅ^{-3}	2.41/-1.87

Supplementary Table 2. Crystal data and structure refinement for $(AuAg)_{45}$.

Identification code	$(AuAg)_{45}(2,4\text{-}SPhMe_2)_{27}$
Empirical formula	$C_{324}H_{333}Ag_{36}Au_9P_6S_{27}$
Formula weight	10934.34
Temperature/K	100.01(10)
Crystal system	hexagonal
Space group	$P6_3/m$
a/Å	22.8229(3)
b/Å	22.8229(3)
c/Å	35.8912(6)
α/°	90
β/°	90
γ/°	120
Volume/Å^3	16190.5(5)
Z	2
ρ_{calc} g/cm^3	2.243
μ/mm^{-1}	26.733
F(000)	10404.0
Crystal size/mm^3	0.12 × 0.12 × 0.1
Radiation	6.652 to 143.248
2θ range for data collection/°	CuKα (λ = 1.54184)
Index ranges	$-26 \leq h \leq 19$, $-26 \leq k \leq 27$, $-43 \leq l \leq 40$
Reflections collected	58267
Independent reflections	10537 [R_{int} = 0.0677, R_{sigma} = 0.0361]
Data/restraints/parameters	10537/270/553
Goodness-of-fit on F^2	1.127
Final R indexes [$I \geq 2\sigma$ (I)]	R_1 = 0.0830, wR_2 = 0.1994
Final R indexes [all data]	R_1 = 0.1120, wR_2 = 0.2287
Largest diff. peak/hole / eÅ^{-3}	2.94/-3.00

Supplementary Table 3. A summary of average bond lengths of important bonds in plasmonic $(AuAg)_{267}$ nanoparticle.

Nanoparticle / Bond Length(Å)	$(AuAg)_{267}$	$(AuAg)_{45}$
**Ag(0)-M(1)**	2.685	——
**M(1)-M(1)**	2.822	2.839
**M(1)-M(2)**	2.804	3.003
**M(2)-M(2)**	2.889	3.161
**M(2)-M(3)**	2.865	——
**M(3)-M(3)**	2.915	——
**M(3)-M(4)**	2.907	——
**Ag(4)-Ag(4)**	3.112	——
**Ag(2)-Ag(P)**	——	3.754
**Ag-P**	——	2.416
**Ag-S**	2.662	2.566

Ag(0)-M(1) bond length between central Ag atom and 1st shell.
M(1)-M(1) bond length in the 1st shell.
M(1)-M(2) bond length between the 1st and 2nd shell.
M(2)-M(2) bond length in the 2nd shell.
M(2)-M(3) bond length between the 2nd and 3rd shell.
M(3)-M(3) bond length in the 3rd shell.
M(3)-M(4) bond length between the 3rd and 4th shell.
M(4)-M(4) bond length in the 4th shell.
Ag–P bond length between Ag and phosphine.
Ag–S bond length between Ag and thiolate.
All other data are from structures determined by single-crystal analysis.

Supplementary Table 4. Calculated charges per atom/ligand for the atomic shells of $Au_{85}Ag_{182}(SR)_{80}$ cluster.

Atomic shells	Ntot	N_{Au} / N_{Ag}	Charge/Au-atom |e|	Charge/Ag-atom |e|	Charge / Ligand |e|
**Central atom**	1	0 / 1	-	+0.115	-
**1st shell**	12	7 / 5	-0.092	+0.135	-
**2nd shell**	42	29 / 13	-0.106	+0.167	-
**3rd shell**	92	49 / 43	-0.176	+0.107	-
**4th shell**	120	0 /120	-	+0.251	-
**Ligands**	80	- / -	-	-	-0.317

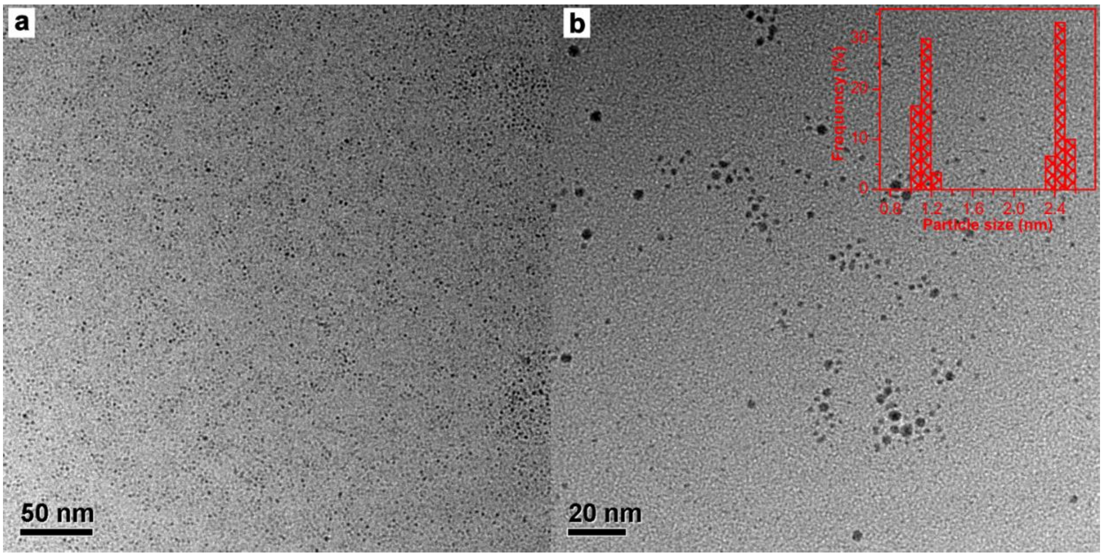

Supplementary Figure 1. TEM images of $(AuAg)_{267}$·$(AuAg)_{45}$ with different magnifications. The scale bars in (a) and (b) are 50 and 20 nm, respectively. The inset of (b) plot shows the size distribution of the nanoparticles observed by TEM.

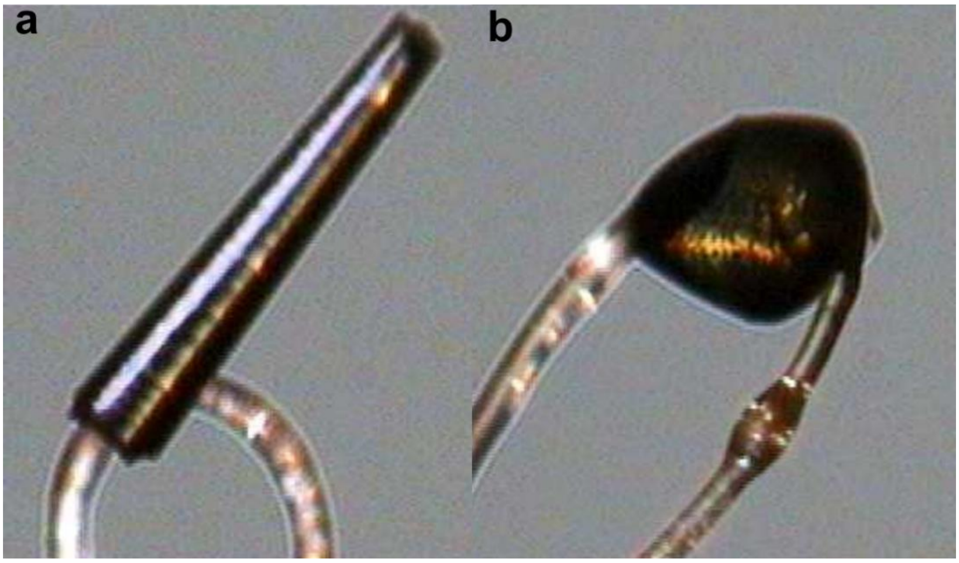

Supplementary Figure 2. Crystal photos of $(AuAg)_{267}$·$(AuAg)_{45}$ (a) and $(AuAg)_{45}$ (b).

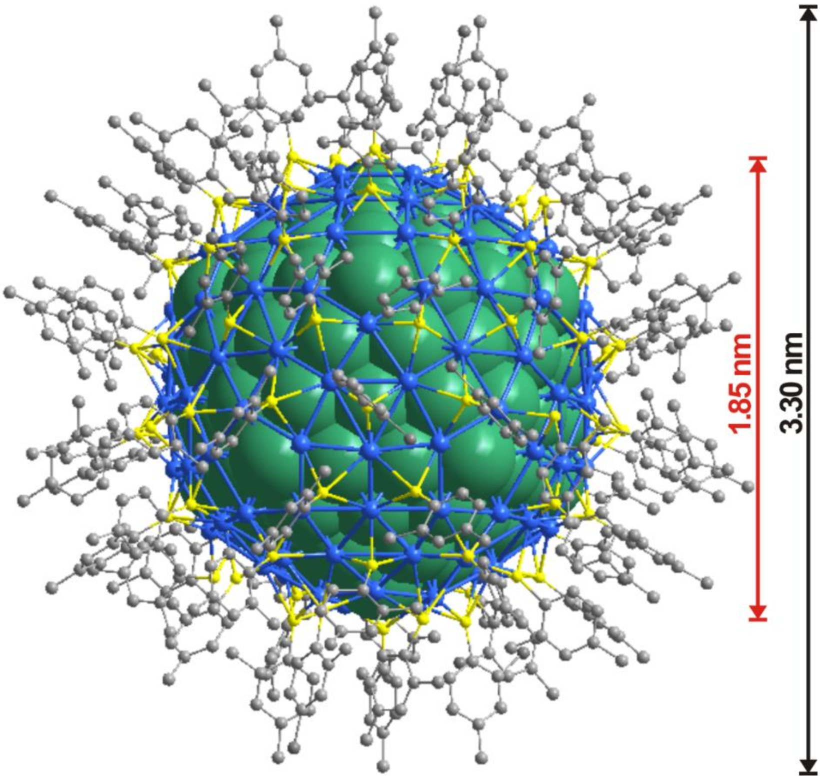

Supplementary Figure 3. Size diagram of whole $(AuAg)_{267}$ nanoparticle and its metal core.

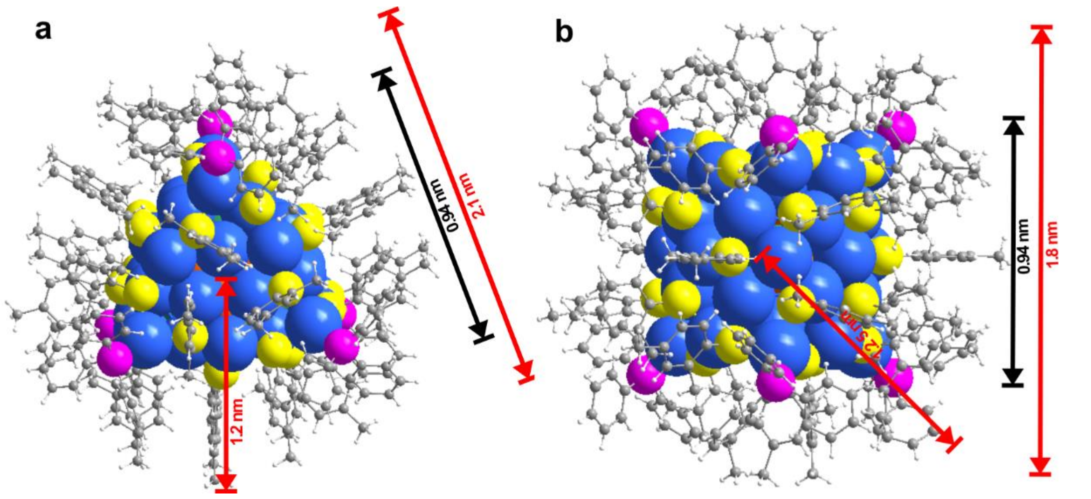

Supplementary Figure 4. Size diagram of whole $(AuAg)_{45}$ nanocluster and its metal core. Top view (a) and side view (b).

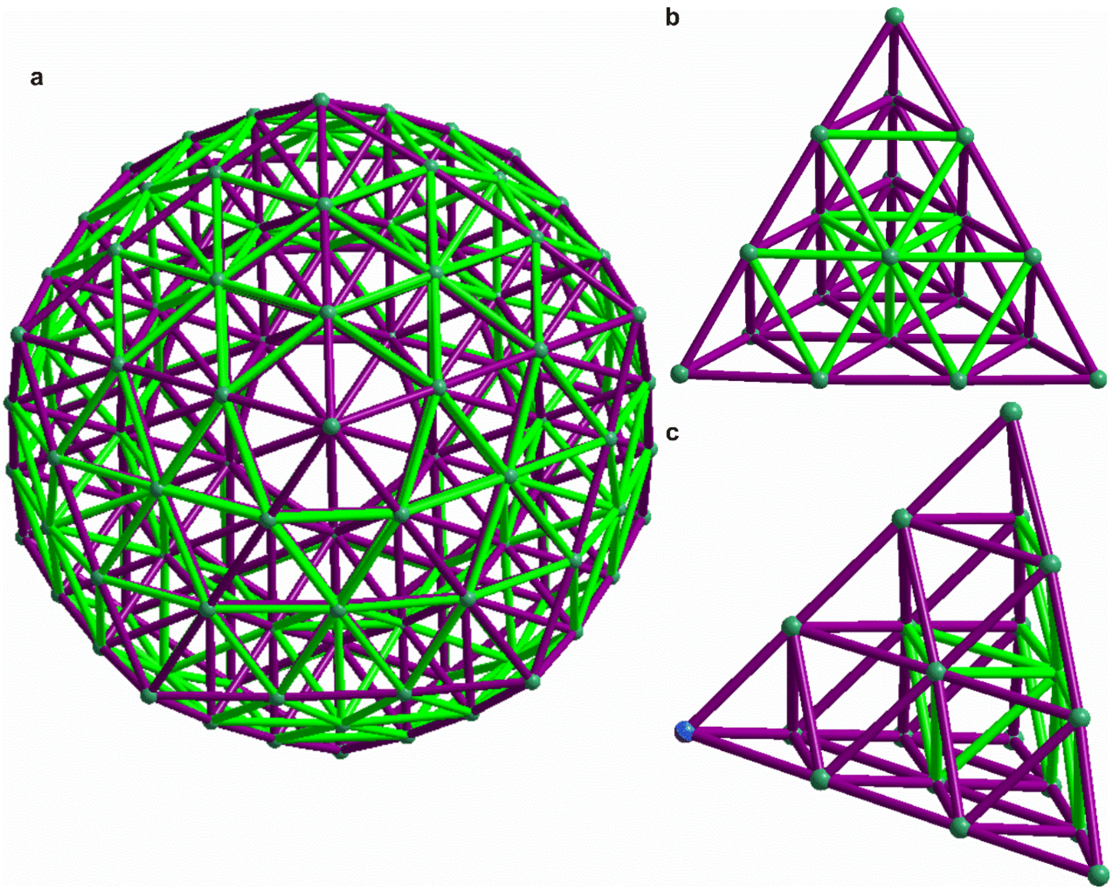

Supplementary Figure 5. Bond analysis of metals at the $(AuAg)_{147}$ core of plasmonic $(AuAg)_{267}$ nanoparticle. The $(AuAg)_{147}$ icosahedral core (a); top view (b) and side view (c) of its $(AuAg)_{20}$ tetrahedral subunit. Yellow and purple bonds are shorter than the average bond distance (2.877 Å), light green Ag-Ag are longer than 2.877 Å. Color codes: sea green, AuAg; blue, Ag; All hydrogen, sulphur and chlorine atoms are omitted for clarity.

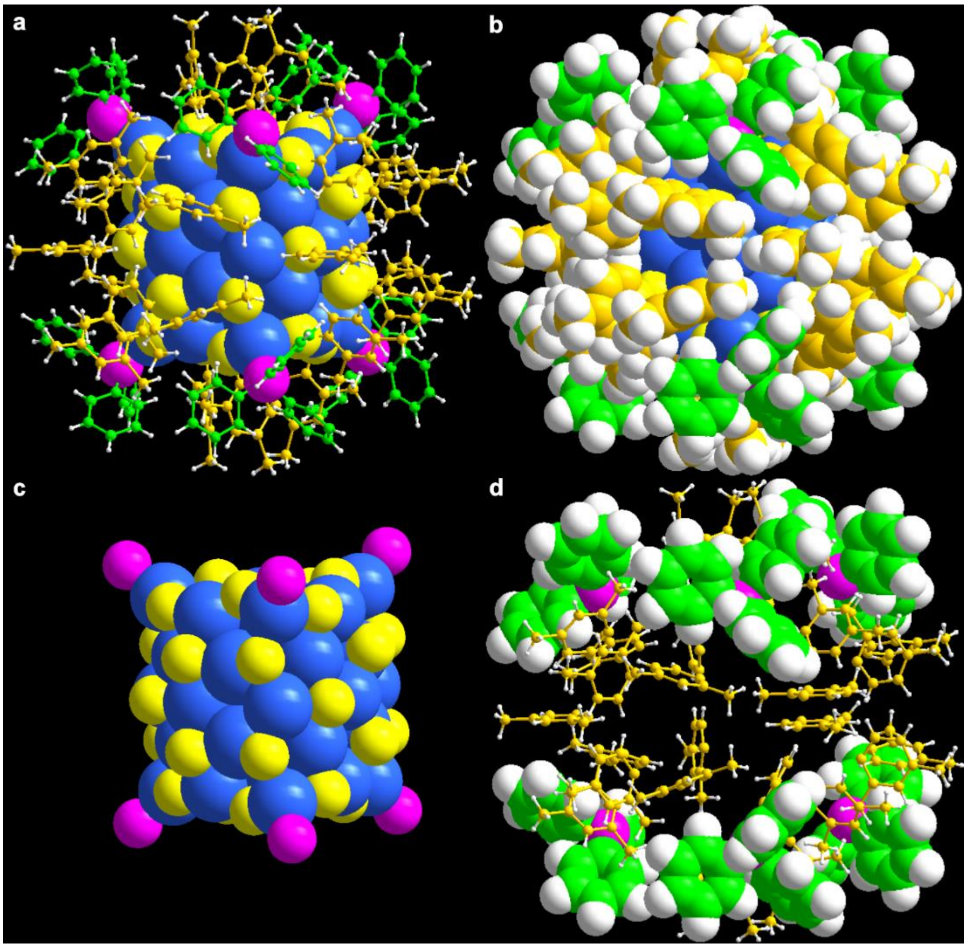

Supplementary Figure 6. Anisotropic surface patterns of the ligands on the $(AuAg)_{45}$ cluster. Overall structures of ligands on the surface of cluster in ball-and-stick (a) and space-filling (b) model. (c) Tri-prism core of the cluster. (d) Anisotropic ligand shell (phosphines in space-filling model and thiolate in ball-and-stick model). Color code: blue, Ag and Au; yellow, S; magenta, P; gold and green, C; white, H.

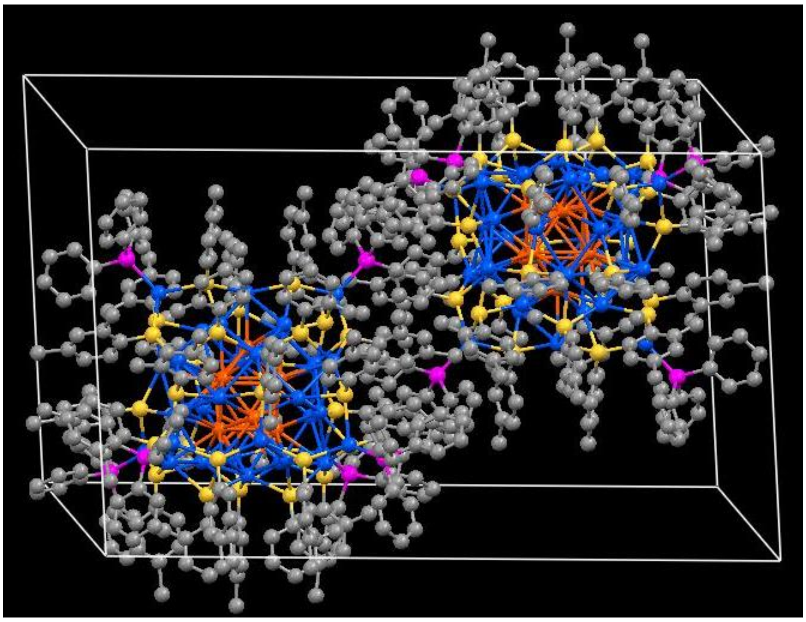

Supplementary Figure 7. Unit cell of the three-dimensional structure of the pure $(AuAg)_{45}$ crystal. All hydrogen and carbon atoms are omitted for clarity. Color code: orange, Au; blue, Ag; yellow, S; magenta, P; grey, C.

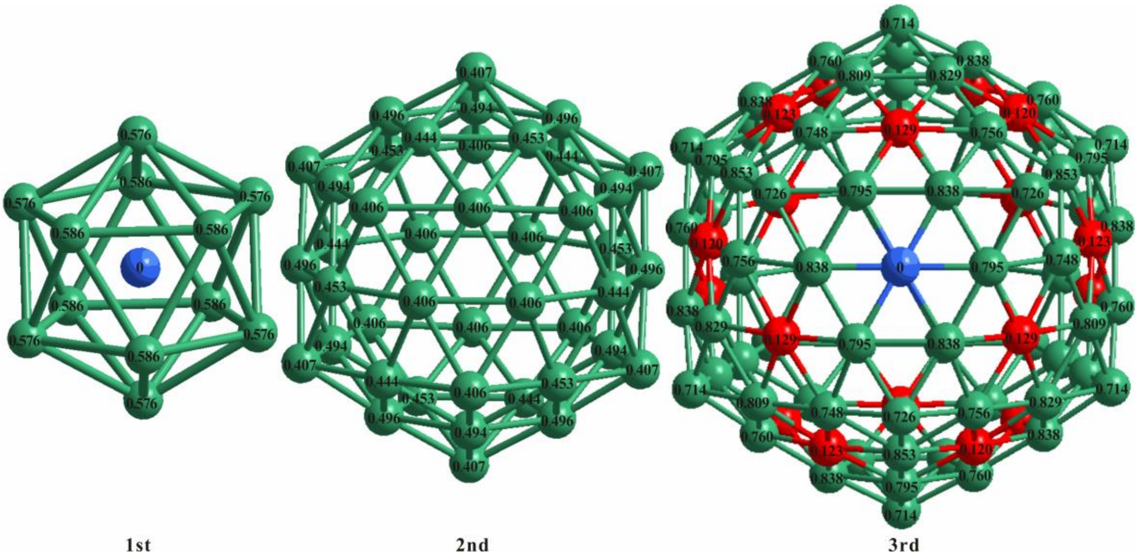

Supplementary Figure 8. Au-Ag-Atom distribution regularities in $(AuAg)_{267}$ nanoparticles based upon least-squares refinement of the X-ray data. The 147-atom icosahedral kernel of $(AuAg)_{267}$ nanoparticle. Notely, number codes represent different crystallographic metal occupancy of gold in $(AuAg)_{267}$ alloy compound. Colour code: sea green or red, AuAg; blue, Ag.

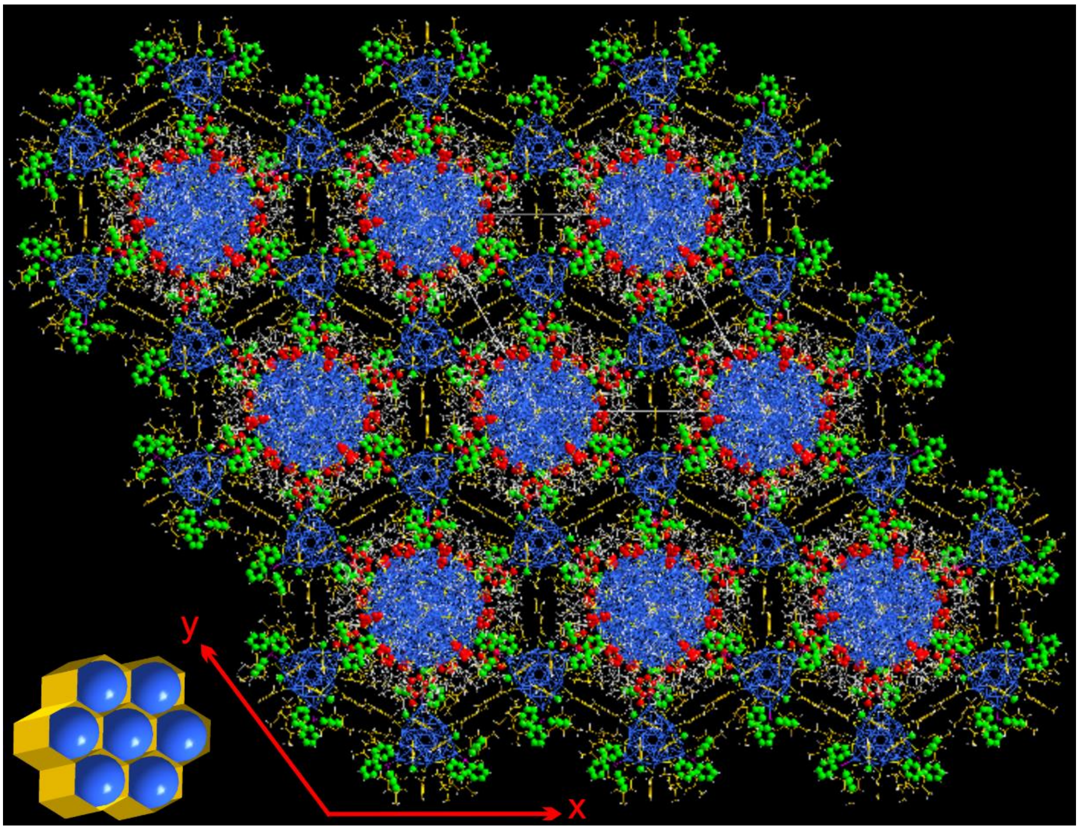

Supplementary Figure 9. The self-assembly packing structure of plasmonic $(AuAg)_{267}$ nanoparticle and molecule-like $(AuAg)_{45}$ clusters (view from z direction). Inset is the schematic diagram of hive structure. Carbon atoms in phosphine highlight in green, thiolate of $(AuAg)_{45}$ highlight in gold, thiolates of $(AuAg)_{267}$ highlight in grey except those forming C-H···π interactions with lateral cluster in red. Color code: blue, Ag and Au; yellow, S; magenta, P; grey, red, gold and green, C; white, H.

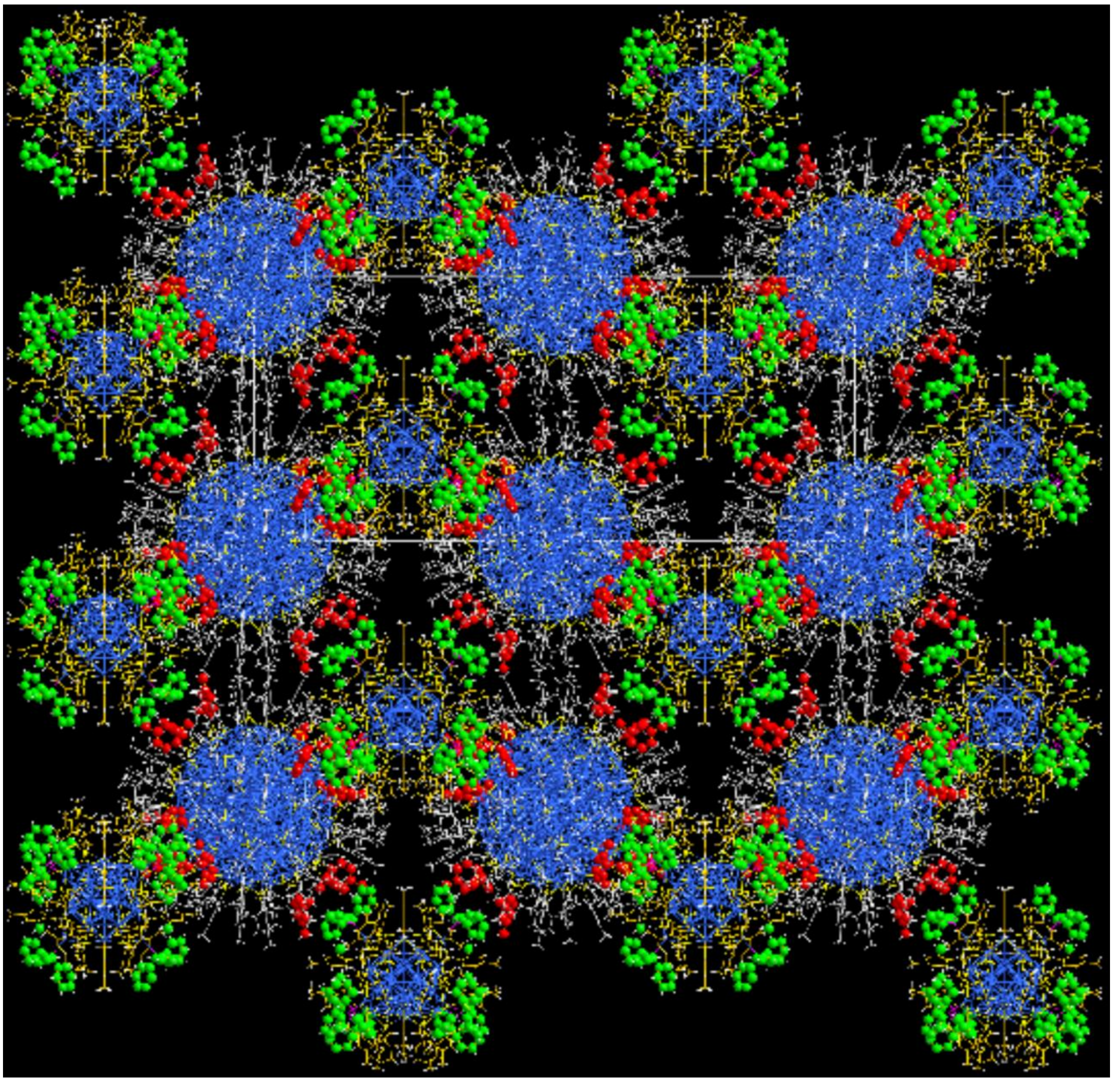

Supplementary Figure 10. The self-assembly packing structure of plasmonic $(AuAg)_{267}$ nanoparticle and molecule-like $(AuAg)_{45}$ clusters (view from x direction). Carbon atoms in phosphine highlight in green, thiolate of $(AuAg)_{45}$ highlight in gold, thiolates of $(AuAg)_{267}$ highlight in grey except those forming C-H⋯π interactions with lateral cluster in red. Color code: blue, Ag and Au; yellow, S; magenta, P; grey, red, gold and green, C; white, H.

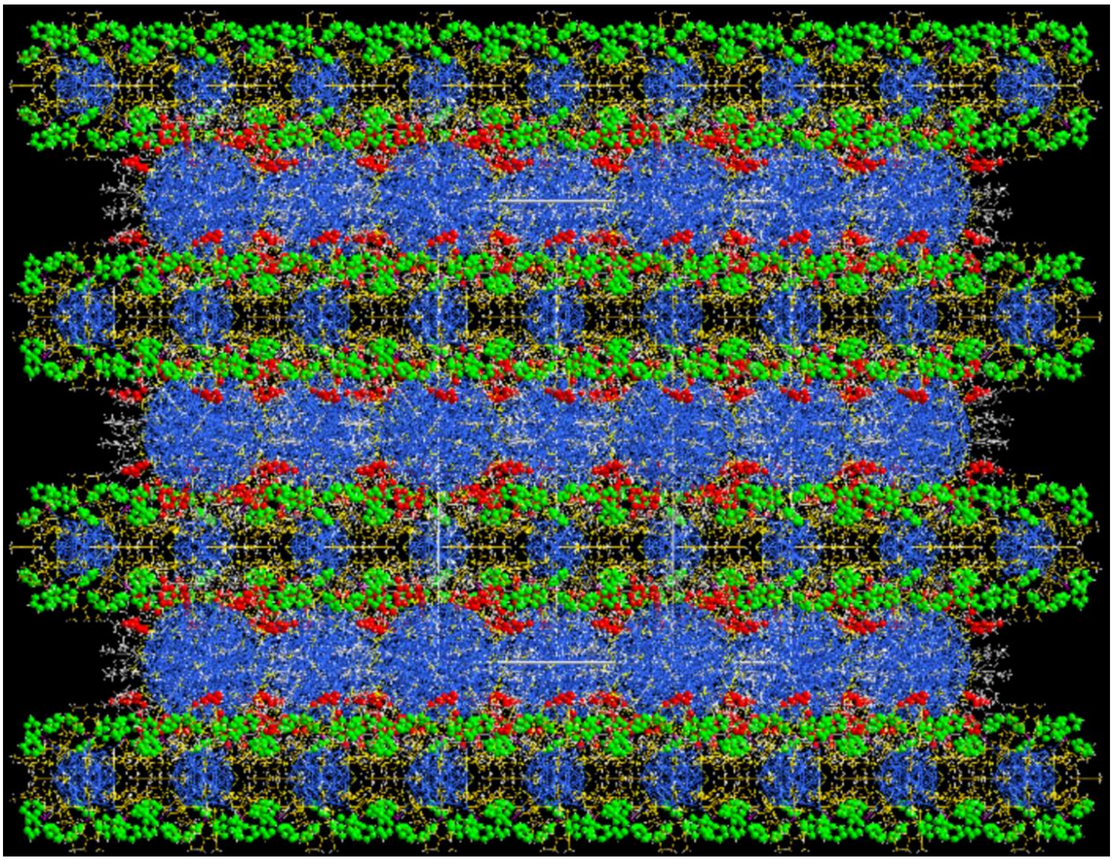

Supplementary Figure 11. The self-assembly packing structure of plasmonic $(AuAg)_{267}$ nanoparticle and molecule-like $(AuAg)_{45}$ clusters (view from y direction). Carbon atoms in phosphine highlight in green, thiolate of $(AuAg)_{45}$ highlight in gold, thiolates of $(AuAg)_{267}$ highlight in grey except those forming C-H···π interactions with lateral cluster in red. Color code: blue, Ag and Au; yellow, S; magenta, P; grey, red, gold and green, C; white, H.

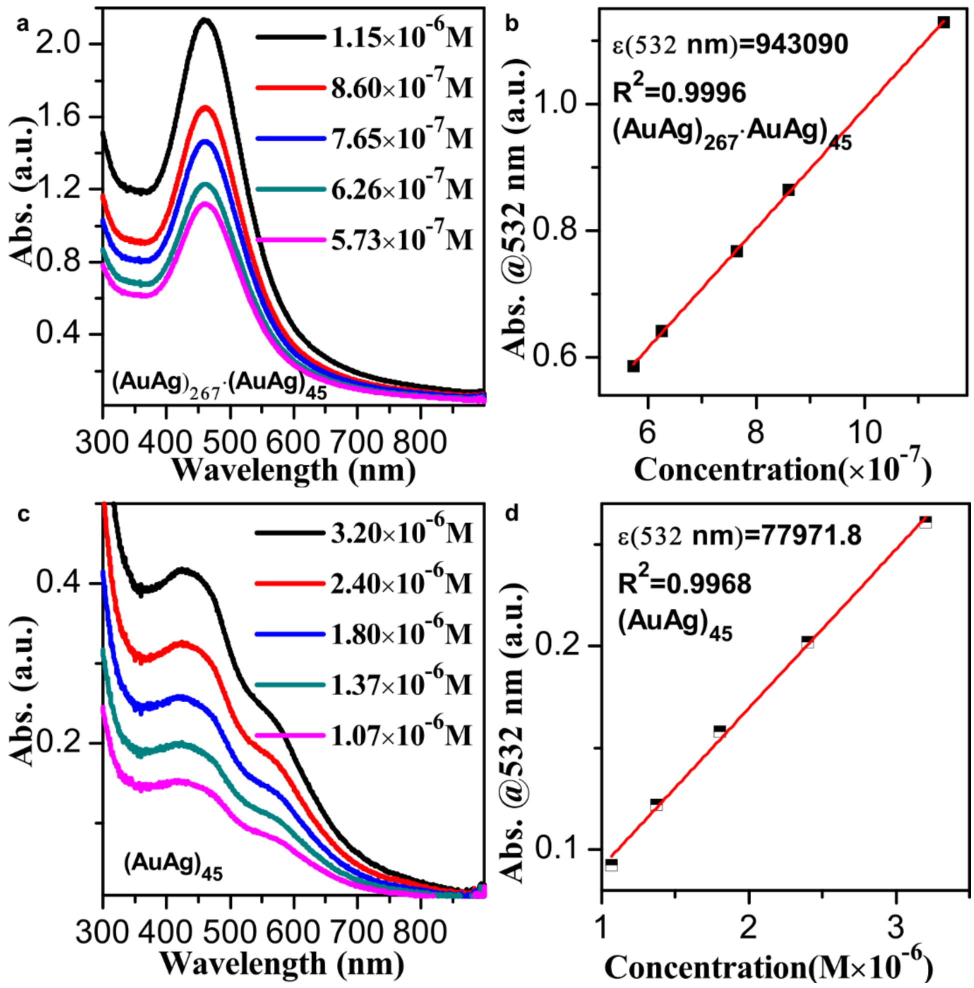

Supplementary Figure 12. Molar absorptivity measurement of $(AuAg)_{267}$·$(AuAg)_{45}$ and $(AuAg)_{45}$. (a) UV-vis spectra of $(AuAg)_{267}$·$(AuAg)_{45}$ dissolved in dichloromethane. (b) calibration plot for determination of $(AuAg)_{267}$·$(AuAg)_{45}$; λ=532 nm, R^2 = 0.9996, molar absorptivity (ε) = 943090 cm^{-1}·M^{-1}. (c) UV-Vis spectra of $(AuAg)_{45}$ dissolved in dichloromethane. (d) Calibration plot for determination of $(AuAg)_{45}$; λ = 532 nm, R^2 = 0.9968, molar absorptivity (ε) = 77971.8 cm^{-1}·M^{-1}.

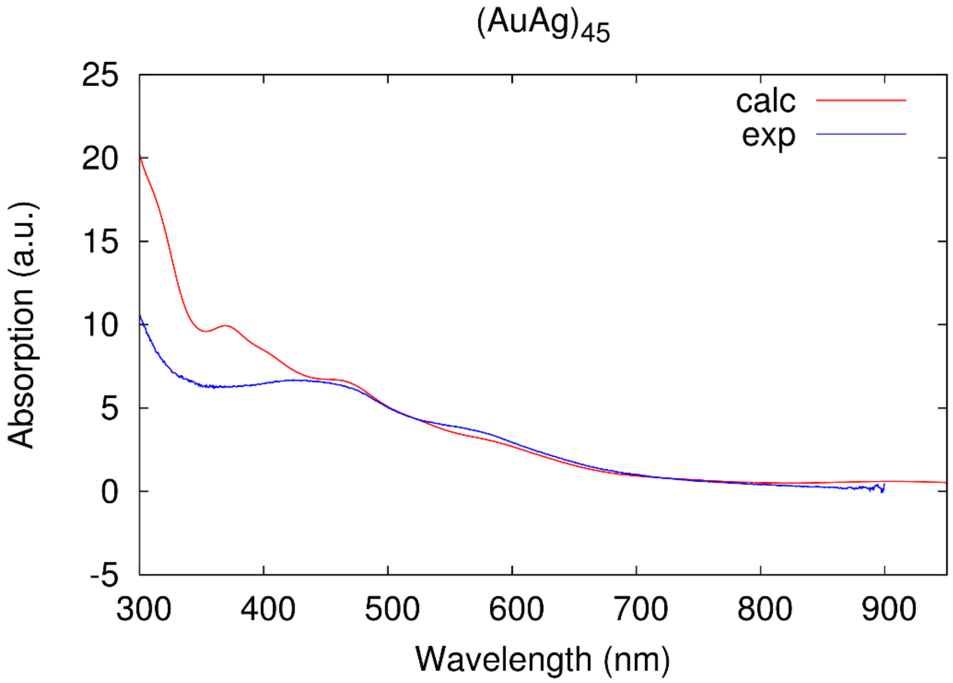

Supplementary Figure 13. Comparison of calculated and experimental UV-vis spectra of $(AuAg)_{45}$ cluster.

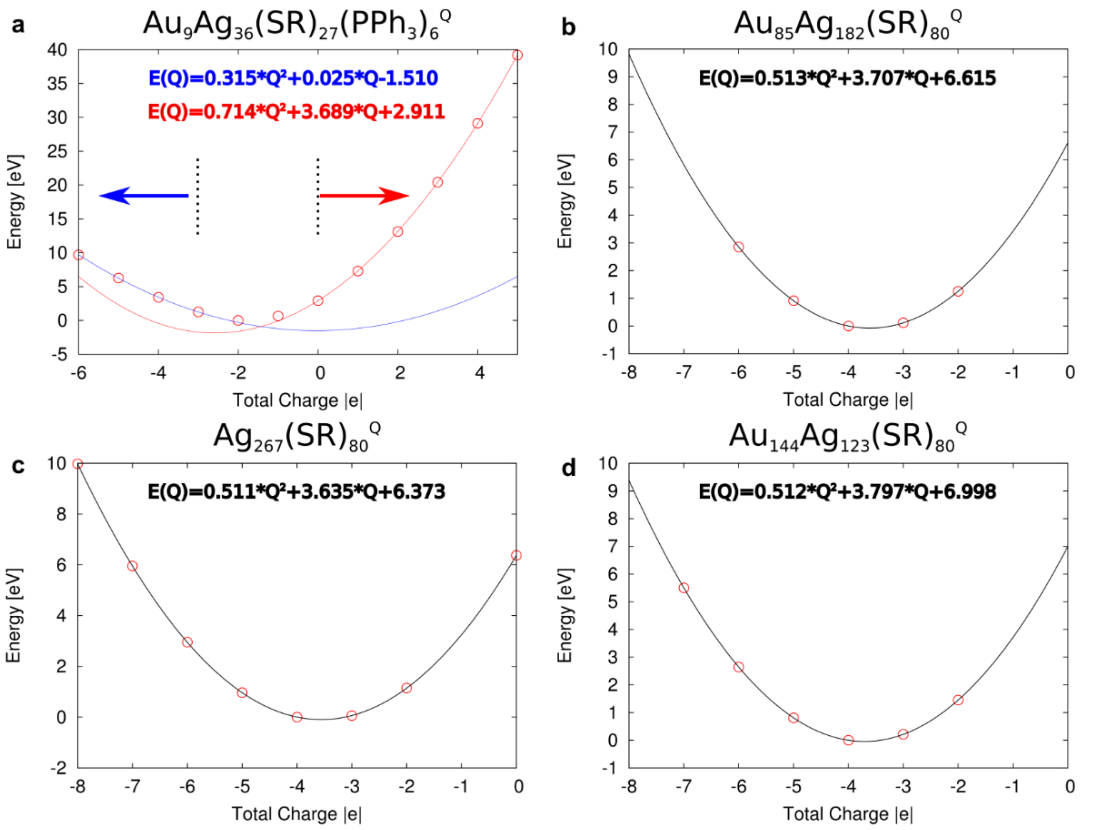

Supplementary Figure 14. Charging behavior of the clusters in the co-crystal. (a) DFT-computed total energy as a function of the cluster charge, $E(Q)$, of the $(AuAg)_{45}$ cluster. (b-d) The same results for the all-silver Ag_{267} and for two intermetallic clusters with compositions of $Au_{85}Ag_{182}$ and $Au_{144}Ag_{123}$, all computed in the observed crystal structure of $(AuAg)_{267}$. The results are fitted to the parabolic theory of charging of a metallic sphere, where $E(Q)$ is expected to behave quadratically. In (a), two separate fits are shown, corresponding to filling of the nearly degenerate electron states $Q = 0$ and $Q = 4$ (red curve), and the other one for filling the states between $Q = -6$ and $Q = -3$ (blue curve). A zoom-in of (b) in the region of the HOMO-LUMO gap is shown in Supplementary Figure 15.

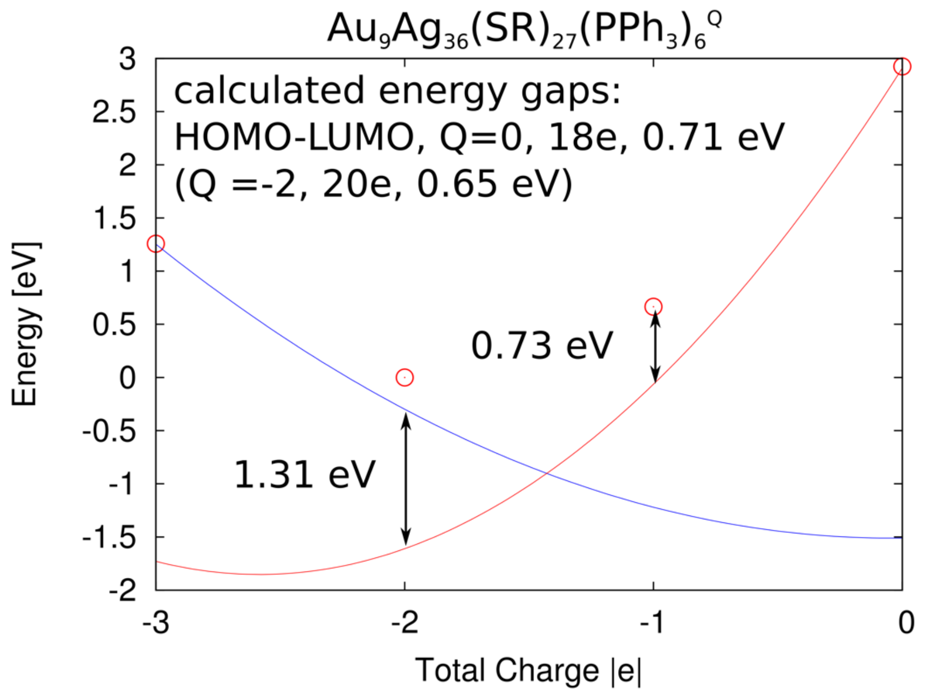

Supplementary Figure 15. Zoom-in of Supplementary Fig. 14a in the HOMO-LUMO gap region. The quadratic fits are the same as shown in Supplementary Fig. 14. Note that the difference between the red curve and the calculated energy at Q = -1 (0.73 eV) is very close to the actual HOMO-LUMO gap of $(AuAg)_{45}$ cluster (0.71 eV), and the difference between the predicted energy curves at Q = -2 (1.31 eV) is close to the sum of quantum energy gaps at 18 and 20 electrons (0.71 eV + 0.65 eV = 1.36 eV).

Supplementary References

1. Yamamoto, Y., Nishina, N. Gold-Catalyzed Intermolecular Hydroamination of Allenes: First Example of the Use of an Aliphatic Amine in Hydroamination. *Synlett* 1767-1770 (2007).

2. CrysAlis^{Pro} Version 1.171.35.19. (2011). Agilent Technologies Inc. Santa Clara, CA, USA.

3. Sheldrick, G. M. SHELXT-Integrated space-group and crystal-structure determination. *Acta Cryst.* **A71**, 3-8 (2015).

4. Sheldrick, G. M. A short history of SHELX. *Acta Cryst*. **A64**, 112-122 (2008).

5. Dolomanov, O. V.; Bourhis, L. J.; Gildea, R. J.; Howard, J. A. K.; Puschmann, H. OLEX2: a complete structure solution, refinement and analysis program. *J. Appl. Cryst*. **42**, 339-341 (2009)..

6. Hübschle C. B., Sheldrick, G. M., Dittrich B. ShelXle: a Qt graphical user interface for SHELXL. *J. Appl. Cryst.*, **44**, 1281-1284 (2011).
